# Utilizing machine learning to predict post-treatment outcomes in chronic non-specific neck pain patients undergoing cervical extension traction

**DOI:** 10.1038/s41598-024-62812-7

**Published:** 2024-05-23

**Authors:** Ibrahim M. Moustafa, Dilber Uzun Ozsahin, Mubarak Taiwo Mustapha, Amal Ahbouch, Paul A. Oakley, Deed E. Harrison

**Affiliations:** 1 Department of Physiotherapy, College of Health Sciences, University of Sharjah, 27272 Sharjah, United Arab Emirates; 2 Neuromusculoskeletal Rehabilitation Research Group, RIMHS–Research Institute of Medical and Health Sciences, University of Sharjah, 27272 Sharjah, United Arab Emirates; 3 Faculty of Physical Therapy, Cairo University, Giza, 12613 Egypt; 4 Department of Medical Diagnostic Imaging, College of Health Science, University of Sharjah, Sharjah, United Arab Emirates; 5 Operational Research Centre in Healthcare, Near East University, TRNC Mersin 10, 99138 Nicosia, Turkey; 6 Research Institute for Medical and Health Sciences, University of Sharjah, Sharjah, United Arab Emirates; 7 Department of Biomedical Engineering, Near East University, Nicosia, Mersin 10, Turkey; 8 CBP Nonprofit (a Spine Research Foundation), Eagle, ID 83616 USA; 9 Private Practice, Newmarket, ON L3Y 8Y8 Canada; 10 Kinesiology and Health Science, York University, Toronto, ON M3J 1P3 Canada

**Keywords:** Cervical spine, Lordosis, Traction, Neck pain, Disability, Prediction, Machine learning, Health care, Medical research

## Abstract

This study explored the application of machine learning in predicting post-treatment outcomes for chronic neck pain patients undergoing a multimodal program featuring cervical extension traction (CET). Pre-treatment demographic and clinical variables were used to develop predictive models capable of anticipating modifications in cervical lordotic angle (CLA), pain and disability of 570 patients treated between 2014 and 2020. Linear regression models used pre-treatment variables of age, body mass index, CLA, anterior head translation, disability index, pain score, treatment frequency, duration and compliance. These models used the sci-kit-learn machine learning library within Python for implementing linear regression algorithms. The linear regression models demonstrated high precision and accuracy, and effectively explained 30–55% of the variability in post-treatment outcomes, the highest for the CLA. This pioneering study integrates machine learning into spinal rehabilitation. The developed models offer valuable information to customize interventions, set realistic expectations, and optimize treatment strategies based on individual patient characteristics as treated conservatively with rehabilitation programs using CET as part of multimodal care.

## Introduction

Neck pain is a major contributor to the global burden of disease and is rated as the 4th greatest contributor to global disability^1^. Chronic neck pain is associated with reduced productivity and increased healthcare utilization, and can lead to functional impairment and psychological distress; both of which can compromise overall quality of life^2^. Although psychosocial factors are important in recognizing characteristics associated with developing neck pain^3,4^, it has been shown that studies evaluating the contributions of these factors to chronic musculoskeletal pain lacks quality and fails to explain a significant remaining proportion of the statistical variance^5,6^.

There is a growing interest concerning the understanding of the biomechanics of the sagittal configuration of the cervical spine^7–9^. Importantly, in the past 2 decades, cervical sagittal alignment has gained more attention as an important clinical outcome in healthcare. It has been quite extensively demonstrated that biomechanical dysfunction represented in abnormal cervical sagittal alignment significantly influences human health and well-being, as it has been shown to be associated with pain^10–12^, disability^12,13^, overall functional performance^14^, and ultimately, quality of life^15^.

Documentation of normative limits for sagittal spine alignment parameters has enabled both spine rehabilitation specialists and surgeons to establish standardized goals. These benchmarks play a crucial role in facilitating comparisons during both pre- and post-treatment decision-making strategies, offering a valuable reference point for assessing and optimizing patient outcomes^12,16–23^. Furthermore, ongoing research has led to the development of conservative corrective approaches focused on restoring the normal sagittal plane alignment, with early findings showing significant promise in these approaches^22–24^. These preliminary randomized clinical trials (RCT’s) demonstrated that patients experiencing improvements in lordosis through various types of cervical extension traction (CET) maintained these benefits with relative stability, with minimal loss at 3-months to 2-year follow-ups^22,23^. Notably, these RCT’s documented enhanced pain^23,24^, disability^23,24^, and neurophysiology^22,23^ alongside improvements in cervical lordosis in contrast to non-lordotic changed comparative treatment groups where limited improvements were observed^22–24^.

Technological advances in both diagnosis and treatment for patients suffering from spine pain and disability are necessary to reduce costs, improve outcomes, increase efficiency for the facilities and clinicians as well as reduce poor outcomes and complications that may require additional care; these statements are true for both conservative and surgical interventions for spine disorders^25–29^. Technology creates a more efficient and efficacious process of spine condition diagnosis and treatment, however, while machine learning has demonstrated considerable success in various medical applications^25–29^, its implementation in predicting rehabilitation outcomes after a multimodal rehabilitative care program designed to improve cervical sagittal malalignment remains an underexplored area. Thus, the current research seeks to bridge this gap by applying advanced machine learning techniques to design predictive models for rehabilitation outcomes of patients undergoing a multimodal program of care to improve chronic neck pain and related disability. Building on the previous groundwork^22–24^, in this study, machine learning techniques were applied to gain further insight into the role of pre-treatment factors in predicting outcomes for patients experiencing chronic neck pain undergoing a multimodal care program incorporating CET methods to improve sagittal cervical alignment. The research methodology focuses on pre-treatment factors, including demographic information, sagittal cervical alignment, and other pertinent clinical variables, with the explicit goal to predict improvements in the cervical lordotic angle, pain levels, and functional disability status that occur following intervention.

## Results

### Patient characteristics

**Figure 1:**
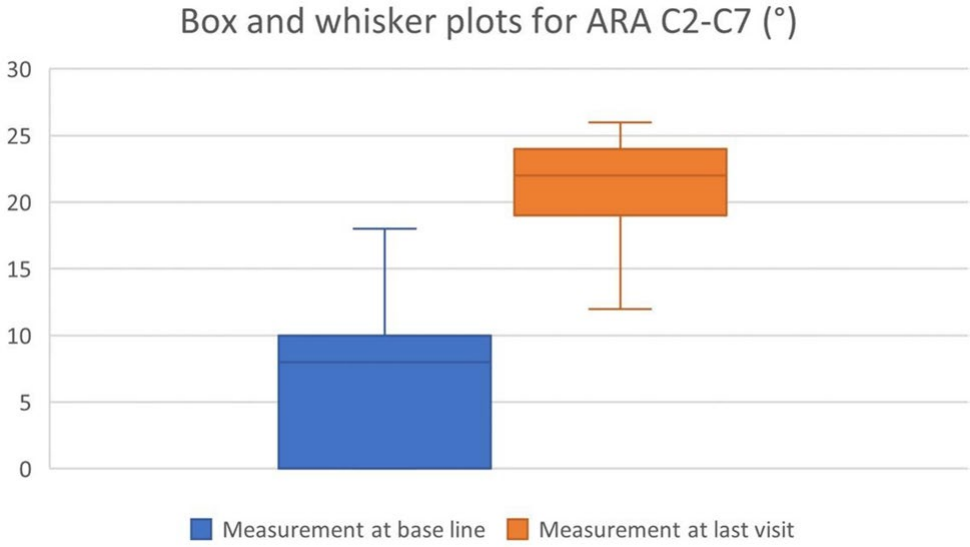
Box and whisker plots for ARA C2–C7 (°) at baseline and final evaluation.

**Figure 2:**
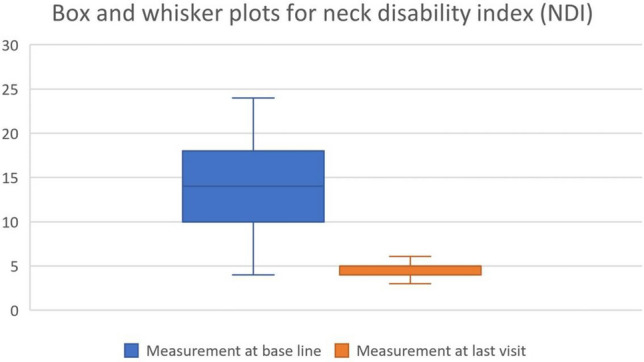
Box and whisker plots for neck disability index (NDI) at baseline and final evaluation. Presented as raw score out of 50 and percentage disability is multiplied by 2.

**Figure 3:**
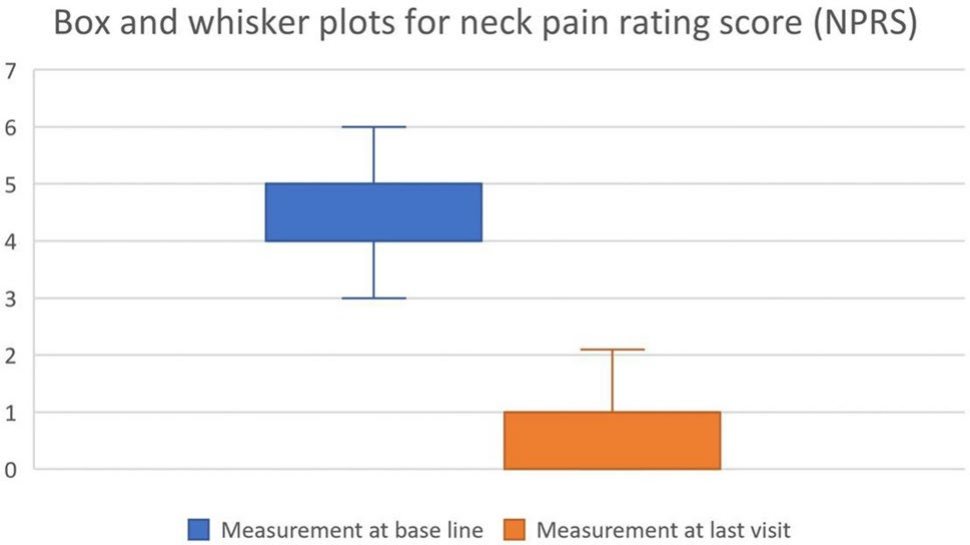
Box and whisker plots for neck pain rating score (NPRS) intensity score from 0 to 10 at baseline and final evaluation.

Details regarding patient characteristics can be observed in Tables 1, 2 and Figs. 1, 2, 3, each of which provides scores at baseline and the final re-evaluation session for all individuals. The cervical lordotic absolute rotation angle (ARA C2–C7) showed a significant increase post-treatment, indicating improvement, with a large effect size (Cohen’s d = 3.11). The follow-up data for anterior head translation (AHT) was not available in the dataset for comparison. The NDI (%) showed a substantial decrease, indicating a reduction in disability, with a large negative effect size (Cohen’s d = − 2.64), suggesting good improvement where 82.1% and 71.4% of the patients achieved a minimal clinically important difference (MCID) of 5.5 and 6 points, respectively, or greater. The pain score (NPRS) also showed a substantial decrease following intervention, indicating a reduction in pain severity, with a large negative effect size (Cohen’s d = − 4.37), suggesting significant improvement where 96.7% of patients achieved an MCID of 2 points or greater. Table 2 presents this data.

**Table 1:** Patient demographics and characteristics (n = 570).

Variables	Measurement at base line
Frequency of traction (no. per week)	4.1 ± 1.2
Duration of traction (no. of weeks)	6.8 ± 2
Age (years)	37.3 ± 9.18; range 19–61
BMI (kg/m^2^)	24.9 ± 4.8
Compliance (%)	92.1 ± 5.4

Data is presented as means ± standard deviations. Number (No.) of tractions treatments per week and number of weeks total. Age; body mass index (BMI); Compliance is reported as the percentage (%) of kept versus scheduled appointments.

**Table 2:** Means, standard deviation, and Cohen’s d for the radiographic measurements (cervical lordosis (ARA C2–C7), anterior head translation (AHT)), neck disability index (NDI), and numerical pain rating score (NPRS).

Variable	Baseline mean (SD)	Follow-up mean (SD)	Cohen’s d
ARA C2–C7 (°)	6.1 ± 5.35	20.86 ± 4.09	3.11
AHT (mm)	22.25 ± 6.8	NR	NR
NDI (raw score) % disability	13.96 ± 4.81; range (8–48%)	4.42 ± 1.73 Achieved MCID: 82.1% (5.5 points) and 71.4% (6 points)	− 2.64
Pain score (NPRS)	4.4 ± 0.80; range (2–7)	0.85 ± 0.69 Achieved MCID: 96.7%	− 4.37

NDI raw score is the total out of 50 possible. Data at follow-up for the NPRS and NDI are presented as the percentage (%) of patients achieving the minimal clinical important difference (MCID) of at least 5.5 and 6 points for the NDI and 2 points for the NRS.

*NR* not reported.

### Model development and specification

In Table 3, the linear regression results for the cervical lordotic angle [C2–C7 absolute rotation angle (ARA)], neck disability index (NDI), and numerical rating scale (NPRS) for pain score are shown. The significance of each metric is as follows:

1. Mean squared error (MSE):
  Lordotic angle: The MSE for the lordotic angle is 6.628, indicating the average squared difference between the predicted and actual values. A lower MSE suggests better model precision in predicting the post-treatment ARA.
  Disability index: The MSE of 2.091 for disability index reflects the model's ability to minimize errors in predicting post-treatment NDI scores.
  Pain score: With a MSE of 0.342, the model demonstrates high accuracy in predicting post-treatment NRS pain scores.
2. R-squared:
  Lordotic angle: The R-squared value of 0.549 signifies that the linear regression model can explain approximately 54.9% of the post-treatment ARA variability.
  Disability index: An R-squared of 0.305 indicates that around 30.5% of the variability in the post-treatment NDI is captured by the model.
  Pain score: The R-squared value of 0.298 suggests that the model explains approximately 29.8% of the variability in post-treatment NRS pain scores.

3. Mean absolute error (MAE):
  Lordotic angle: The MAE of 2.083 for the ARA represents the average absolute difference between predicted and actual values, providing a measure of model accuracy.
  Disability index: With an MAE of 1.068, the model exhibits a relatively low average absolute error in predicting the post-treatment NDI.
  Pain score: The MAE of 0.492 indicates the model's accuracy in predicting post-treatment NRS pain scores.
4. Root mean squared error (RMSE):
  Lordotic angle: The RMSE of 0.0158 for ARA is the square root of the MSE and provides a standardized measure of prediction accuracy.
  Disability index: A RMSE of 0.000 for NDI suggests a minimal error in predicting the post-treatment NDI.
  Pain score: Similarly, the RMSE of 0.000 for NRS pain score indicates precise predictions with minimal error.

**Table 3:** Linear regression results for lordotic angle (ARA C2–C7), disability index (NDI), and pain score (NRS).

	Mean squared error	R-squared	Mean absolute error	Root mean squared error
Linear regression for post-treatment ARA cervical lordotic angle	6.628	0.549	2.083	0.0158
Linear regression for post-treatment NDI	2.091	0.305	1.068	0.000
Linear regression for post-treatment NRS pain score	0.342	0.298	0.492	0.000

### Scatter plots

**Figure 4:**
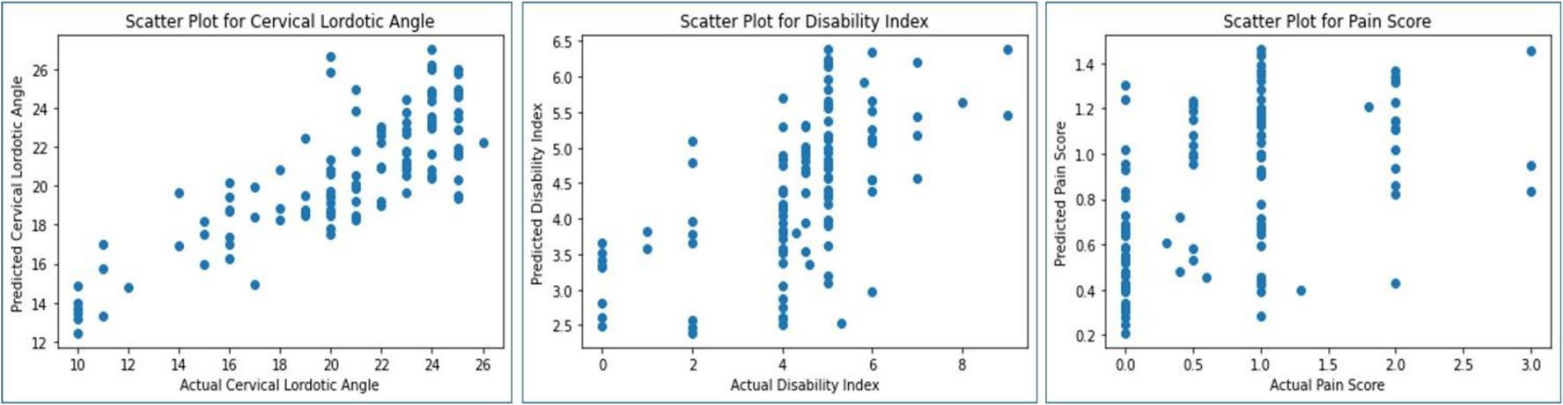
Scatter plot of the predicted vs. actual outcomes for cervical lordotic angle (ARA C2–C7), disability index (NDI), and pain score (NRS). The x-axis is the actual participant’s score for ARA, NDI, NPRS, while the y-axis shows the model predictions.

The scatter plots vividly depict the relationship between the actual and predicted outcomes for the three key variables: (1) ARA or the cervical lordotic angle, (2) the NDI or disability index, and (3) the NPRS pain score as shown in Fig. 4. Each point on the plots represents an observation, with the x-axis indicating the actual values and the y-axis representing the corresponding predictions. In the ARA cervical lordotic angle scatter plot, the points clustering around a diagonal line suggest a strong correlation between actual and predicted angles. Similarly, for the NDI and NPRS, the proximity of points to the diagonal indicates the model's effectiveness in capturing the underlying patterns. Deviations from the diagonal line signify prediction errors, and the extent of dispersion showcases the variability in the model's performance.

### Distribution plot

**Figure 5:**
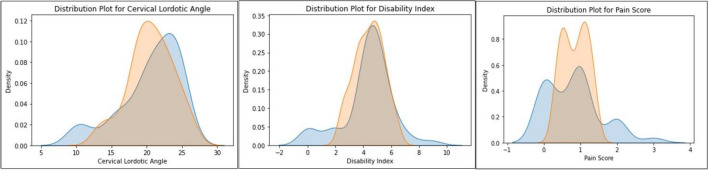
Distribution plot of the predicted vs. actual outcomes of ARA cervical lordotic angle, NDI as a disability index, and the NPRS pain score. In each plot, the blue is the actual patient response to care and the orange is the model predictions. The shaded areas represent the density of the actual and predicted values, showcasing the model's accuracy in capturing the underlying distribution of the data.

The distribution plots in Fig. 5 vividly illustrate the alignment between the actual and predicted outcomes for the ARA cervical lordotic angle, NDI, and NRS pain score. In each plot, the shaded areas represent the density of the actual and predicted values, showcasing the model's accuracy in capturing the underlying distribution of the data. For the ARA, the peaks of the actual and predicted curves coincide, indicating precise predictions. Similarly, the NDI and NPRS pain score plots reveal overlapping distributions, suggesting the model's ability to effectively estimate these parameters. The tight convergence of the actual and predicted curves in all three plots signifies the model's robust performance in replicating the real-world distribution of post-treatment outcomes. This visual analysis provides confidence in the linear regression models' capability to predict cervical treatment outcomes accurately.

### Feature/variables of importance

In Table 4, the pre-treatment ARA emerges as the most influential factor, positively affecting the cervical lordotic angle with a coefficient of 2.78, followed by frequency of CET (1.84) and duration of CET (1.54). Among other factors, age and compliance also contribute significantly to the ARA cervical lordotic angle, albeit with negative coefficients, suggesting their potential to decrease the outcome. The NDI is notably positively influenced by pre-treatment NDI (0.33), ARA (0.42), and negatively influenced by frequency of CET application (− 0.45) and duration of CET (− 0.59). Higher values of these features correspond to a reduction in the NDI. The NRS pain score is negatively influenced by frequency of CET application (− 0.14) and duration of CET (− 0.24), indicating more and frequent sessions have role in mitigating post-treatment pain. This comprehensive analysis of feature importance not only elucidates the individual contributions of each factor but also aids in understanding the intricate dynamics of the CET procedure's effectiveness.

**Table 4:** Feature importance of the variables is shown.

Feature	Coefficient ARA	Coefficient NDI	Coefficient NRS
Pre-treatment ARA	2.781511	0.426464	0.094271
Pre-treatment AHT	− 0.055375	− 0.047616	− 0.016804
Pre-treatment NDI	− 0.447356	0.330291	0.031285
Pre-treatment NRS	0.110037	− 0.061601	− 0.017791
Frequency of CET	1.844923	− 0.452927	− 0.139162
Duration of CET	1.547450	− 0.586511	− 0.240062
Age (years)	− 0.968734	0.101167	0.036640
BMI	0.215935	0.086257	− 0.076573
Compliance %	− 0.429379	− 0.205086	− 0.013744

A positive and larger value influences the outcome of interest by improving its value after treatment; a negative larger value has the affect of resulting in lesser patient improvement response after treatment. *ARA* cervical lordosis from C2–C7, *AHT* anterior head translation C2–C7, *NDI* neck disability index, *NPRS* numerical rating scale pain score, *CET* cervical extension traction application with the DCTO, *BMI* body mass index; Compliance: % of made appointment sessions. The equation for linear regression analysis was: $\Upsilon_{\left( i \right)} = {\dot{\text{f}}}\left( {Xi,\beta } \right) + {\text{e}}i$; $\Upsilon_{\left( i \right)}$ = dependent variable; ${\dot{\text{f}}}$ = function; Xi = independent variable; β = unknown parameter; and ei = error terms.

## Discussion

More and more, machine learning models are being widely developed and utilized in healthcare examination, diagnosis, and treatment decision algorithms in the efforts to reduce costs, systematize cumbersome processes, and develop prediction rules with the goals of improving selection and outcomes of patients undergoing various interventions^25–29^. To our knowledge, no investigations have used machine learning tools to look at the prediction of patient outcomes undergoing a multimodal rehabilitation program using CET to improve the sagittal plane alignment of the cervical spine in patients suffering from chronic non-specific neck pain (CNSNP) disorders. Since surgical^7,9,12,30^ and conservative^10,11,13,22–24^ analysis and rehabilitation interventions of sagittal cervical malalignment represents a large financial burden for societies, machine learning affords a unique area of opportunity to improve understanding, outcomes, and potentially reduce costs. In our investigation, by integrating Python, sci-kit-learn, and NumPy, we facilitated a rigorous and data-driven approach, allowing for a nuanced exploration of predictive modeling in the context of patient outcomes following a standardized treatment approach including CET^22–24^. By leveraging these sophisticated tools, our study aimed not only to forecast post-treatment outcomes (ARA C2–C7, NDI, and NRS) but also to provide a comprehensive understanding of the factors influencing the efficacy of the CET procedure. In the ensuing sections, we discuss the results of the linear regression models, shedding light on their performance and implications for clinical practice.

A groundbreaking aspect of our investigation lies in its pioneering use of machine learning to predict outcomes in CNSNP patients undergoing the CET procedure using a prospectively collected database that was large enough (570 consecutive patients) to develop and test the models. The study's use of linear regression models to predict post-treatment outcomes demonstrates promising precision and accuracy. The R-squared value is crucial in assessing the accuracy of fit of the linear regression model. A higher R-squared value indicates that the model accounts for a larger proportion of the variability in the outcome variable. The identified R-squared values (ranging from 0.298 to 0.549) indicate a moderate to strong explanatory power for the variability in predicting the outcomes in CNSNP intensity (NPRS), neck pain disability (NDI), and the cervical lordotic angle (ARA C2–C7), respectively. The variability explained by the models, provides valuable insights into the predictive capabilities of the linear regression approach. For the cervical lordotic angle, disability index, and pain score, the respective R-squared values imply that a significant portion of the variance in these post-treatment outcomes can be attributed to the predictors included in the models. This suggests that the linear regression models offer meaningful insights into the factors influencing the outcomes of patients undergoing this multi-modal CET application program of rehabilitation.

For the cervical lordotic angle, the mean squared error (MSE) of 6.628 implies that, on average, predicted values deviate by 6.628 units (°) from actual values of curvature change. The R-squared value of 0.549 indicates that the model can explain approximately 54.9% of post-treatment lordotic angle variability, reflecting a moderate to strong association between predictors and observed angles. The precision in predicting the change in cervical lordotic angle may be attributed to the relatively straightforward biomechanical nature of this outcome, where the chosen predictors have a more direct impact on the cervical spine's alignment. We recognize that enhancing the model's predictive accuracy could be possible by adding other important mechanical factors. For instance, biomechanically, the cervical and thoracic spines are interrelated^7,8,12,30^, adding thoracic alignment features such as thoracic kyphosis magnitude^12^, T1 vertebra sagittal slope^12,30^, the thoracic inlet morphology^30^, and the relationship between cervical lordosis and T1 slope (T1slope—ARAC2–C7 ≤ 20°)^7,12^ as input variables presents an important opportunity for enhancing the predictive capabilities of the model. Additionally, adding other spine regions as mechanical features such as spinopelvic parameters may significantly enhance the model’s robustness and accuracy, considering the correlation between the full spine alignment and the cervical lordotic angle^12^.

The cervical lordosis has been reported to have a relatively broad range of normative alignment values ranging from mildly kyphotic to a deep lordosis and is influence by many variables including type of population being investigated^12,31^, measurement methodology^32^, advanced age^12^, and may be influenced by ethnicity^12^. In the current investigation we choose the cutoff for normal versus abnormal cervical lordosis amount of 20° as it has been identified as a statistically significant value with good sensitivity and specificity in separating normal vs. chronic neck pain populations with little to no postural AHT^31^. Furthermore, the 20° cervical lordosis mark (accounting for differences in measurement techniques) is the average value of post treatment outcomes in chronic pain patients undergoing CET procedures and this value seems to be related to improved short-term and long-term pain, disability and neurophysiological findings^22–24^. Finally, in the current prospective population of 570 patients with CNSNP treated with CET, the actual post treatment cervical lordosis reached the 20° magnitude; further evidencing this as a minimum benchmark of rehabilitation care.

According to the model results, the feature that had greatest influence on the amount of post-treatment change in cervical lordotic curvature was the pre-treatment cervical curve magnitude (ARA C2–7), with a feature coefficient (FC) of 2.78. This seems logical in as much as the more abnormal a neck curve is to begin with, it will result in having a positive affect increasing the potential for curve correction following CET application. Furthermore, features that positively influenced the amount of cervical curve change included frequency (FC of 1.84) and duration (FC of 1.54) of CET application; again, this seems logical as the more often you apply a procedure, in theory, the more benefit should occur. In contrast, three features were identified to negatively influence the amount of cervical lordotic change and these included presenting neck disability score (FC of − 0.45), age (FC of − 0.97), and compliance (FC of − 0.43). These negative feature coefficients, suggest that higher initial NDI scores, older age, and a greater lack of adherence to the recommended program all have the potential to decrease the outcome of curve correction. The finding of increased initial neck disability influencing the outcomes of patients suffering from neck injuries and neck pain has been previously documented in the motor vehicle crash collision literature; which adds credibility to our modelling predictions^33,34^.

Regarding the neck disability index or NDI, the minimal detectable change (MDC) for the NDI is 6.9 and the minimal clinically important difference (MCID) for the NDI is 5.5^35^. Herein, we used two thresholds for the MCID of the NDI of 5.5 and 6 points and we identified that 82.1% and 71.4% of our patients achieved these respective MCID values and above after our treatment program. Our analysis indicates that our model exhibits a MSE of 2.091, suggesting relatively low prediction errors in estimating post-treatment NDI scores. The R-squared value of 0.305 signifies that 30.5% of the variability in post-treatment NDI can be explained by the model, indicating a substantial proportion of captured variance. Our comprehensive analysis of feature importance not only elucidates the individual contributions of each factor but also aids in understanding the intricate dynamics of the CET procedure's effectiveness. According to the model results, the features that had the greatest positive influence on the amount of post-treatment change in neck disability were the pre-treatment cervical curve magnitude (ARA C2–7 with FC of 0.42) and the initial disability level (NDI with FC of 0.33). The positive influence of these features (ARA and NDI) indicate more or faster benefit in those patients presenting with a greater loss of cervical curvature and with an initial higher disability level both indicating greater room for improvement. In contrast, the NDI is notably negatively influenced by the treatment variables of frequency, duration, and compliance % (missed sessions) of CET application, with feature coefficients of − 0.42, − 0.56, and − 0.20, respectively. Previously, it has been demonstrated that a greater dose of care, of up to 16–18 sessions results in a greater improvement in patient outcomes in chronic spine related conditions thus validating our current model’s predictions^36,37^.

In terms of the global burden of disease, chronic neck pain is known to be one of the greatest contributors^1^. Underpinning the understanding and outcomes for patients suffering from this disorder is the influence of any specific variable that might be mitigated in order to improve the odds of recovery; obviously, some patient variables can’t be changed (age, sex, etc.) The NPRS has an MDC of 2.6 and the MCID has been reported to be 1.5^35^. We chose a threshold for the MCID of 2 points to indicate the percentage of patients who achieve the MCID at follow-up treatment on the NPRS and we identified that 96.67% of patients achieved this minimal or above amount of improvement. The high percentage of patients achieving MCID in the post treatment response pain score underscores the clinical relevance of this treatment protocol in reducing pain severity using the NPRS pain score. Furthermore, our modelling findings of a low MSE of 0.342 suggests relatively accurate predictions for the change in pain intensity post treatment with CET; while the R-squared value of 0.298 indicates a moderate association between predictors and observed pain score change. Notably, we identified that the NPRS pain score change is negatively influenced by treatment frequency (FC of − 0.139) and duration (FC of − 0.240) of CET application, indicating that the fewer number of interventional sessions over a less amount of time has a negative impact on a patient achieving greater pain improvements. The finding of increasing dose of treatment application in mitigating post-treatment pain has been previously reported^36,37^, however, this is the first time it has been reported in a population receiving a CET procedure.

The observed patterns suggest that input factors included in the models have a more pronounced impact on lordotic angle than on pain and disability index. This could indicate the complexity of pain and disability in CNSNP is influenced by a broader spectrum of variables not fully captured in the current model. For instance, existing research consistently underscores the significant role of cognitive factors in influencing the levels of disability and pain experienced by patients with persistent chronic pain disorders^38^. Moreover, the stress-buffering model posits that social support plays a constructive role in enhancing health outcomes, shielding individuals from the detrimental impacts of stress. Studies have provided evidence supporting the notion that higher levels of perceived social support and justice correlate with reduced pain severity and decreased pain-related disability among individuals dealing with chronic pain-related psychosocial conditions^39^. Furthermore, a recent meta-analysis identified that psychological interventions delivered by physiotherapists were more effective than standard physiotherapy for CNSNP, but the effect sizes were small-to-medium and these investigations did not include an analysis of any RCT’s using CET devices to rehabilitate the cervical lordosis^40^. Consequently, the current project model struggles to capture the entirety of variability in the complex measures of neck disability and neck pain, resulting in lower R-squared values. This highlights the challenges in predicting outcomes that are inherently influenced by diverse and interconnected factors, emphasizing the need for a more comprehensive and nuanced approach in future predictive modeling efforts.

Interestingly, we identified no predictive value for the amount of forward head posture [(FHP) measured as AHT C2–C7 herein] relative to the outcomes of cervical lordosis, NDI and NRS pain intensity and this seemingly contradicts the prevailing literature^7,10–13^. However, in our current sample of patients the mean initial magnitude of FHP was minimal (22.4 mm) and this is below the threshold of 40 mm that has been reported as the optimum cut point that explains increasing NDI and NRS pain scores^7,41^. Furthermore, the small amount of FHP in our population is unlikely to biomechanically affect the magnitude of cervical lordosis^8^; these two reasons likely explain our lack of predictive values for the amount of FHP in our modelling. However, since the integration of machine learning into spinal rehabilitation has emerged as an important tool for advancing personalized healthcare, several of our model’s results are clinically relevant. First, the capacity to predict treatment outcomes based on individual demographic and clinical patient characteristics has implications for selecting the proper CET application parameters. Spinal rehabilitation specialists, equipped with insights into these factors that influence post-treatment changes, can make better-informed decisions regarding treatment plans, enhance the precision of treatment strategies, and potentially enhance patient satisfaction and adherence. Second, understanding the role that patient age, compliance to a defined course of action, treatment frequency and duration needs, coupled with the amount of initial pain and disability at presentation can help mitigate patient confusion and increase patient participation in their healthcare goals with the effort to focus on things that can be controlled by the patient and provider.

As with any study, it is essential to acknowledge the limitations that may influence the generalizability of the findings. The study's focus on a specific patient population with CNSNP and the potential influence of unmeasured or latent variables introduces an element of uncertainty. The acknowledgement of these limitations opens the door to future research endeavors. Additionally, for all pre-treatment evaluations, we relied on a single evaluation (i.e., “snapshot medicine”). To further improve the accuracy of prediction models we could gather pre-treatment information at several time points to obtain more reliable self-reporting information. For example, neck disability or pain intensity diaries over 7 days could be implemented in routine clinical care. Future directions for research may involve additional predictors specific to pain intensity and disability (psycho-social and emotional variables), which showed a slightly lower R-squared value compared to lordotic angle. Additionally, the incorporation of full spine and other spine alignment data along with more advanced machine-learning techniques could help capture non-linear relationships that may be present in the data, further refining future predictive models. Finally, our current investigation did not include medium to long-term follow-up measures for stability of patient improvements over time. However, previous studies suggest that the improvements in cervical spine alignment and consequent pain and disability improvements can last up to 2 years post-rehabilitation, with minimal loss of benefits^5,23,42^. This durability is likely due to the structural changes in cervical alignment induced by the CET treatment, which need to be monitored in future longitudinal studies.

## Conclusion

This pioneering study integrates machine learning into spine care featuring cervical extension traction (CET) in the multimodal rehabilitation of cervical lordosis for managing chronic non-specific neck pain. The linear regression models demonstrated high precision and accuracy, and effectively explained 30–55% of the variability in post-treatment outcomes, the highest for the cervical lordotic angle. The developed models offer valuable information to customize interventions, set realistic expectations, and optimize treatment strategies based on individual patient characteristics as treated conservatively with rehabilitation programs using CET as part of multimodal care. Further research is necessary to refine these types of models for patients suffering from chronic neck pain related disorders.

## Methodology

An analysis utilizing linear models, an in-depth investigation, was conducted on a prospectively collected dataset containing information from 570 consecutive patients who underwent a multimodal care program designed to improve altered sagittal cervical spine alignment using the Denneroll device for CET in two different outpatient treatment centers. The dataset includes a comprehensive range of pretreatment details such as age, body mass index (BMI), compliance, frequency of treatment, duration of traction, NDI for disability, NRS for pain intensity, and sagittal cervical radiographic alignment parameters.

### Participants

**Figure 6:**
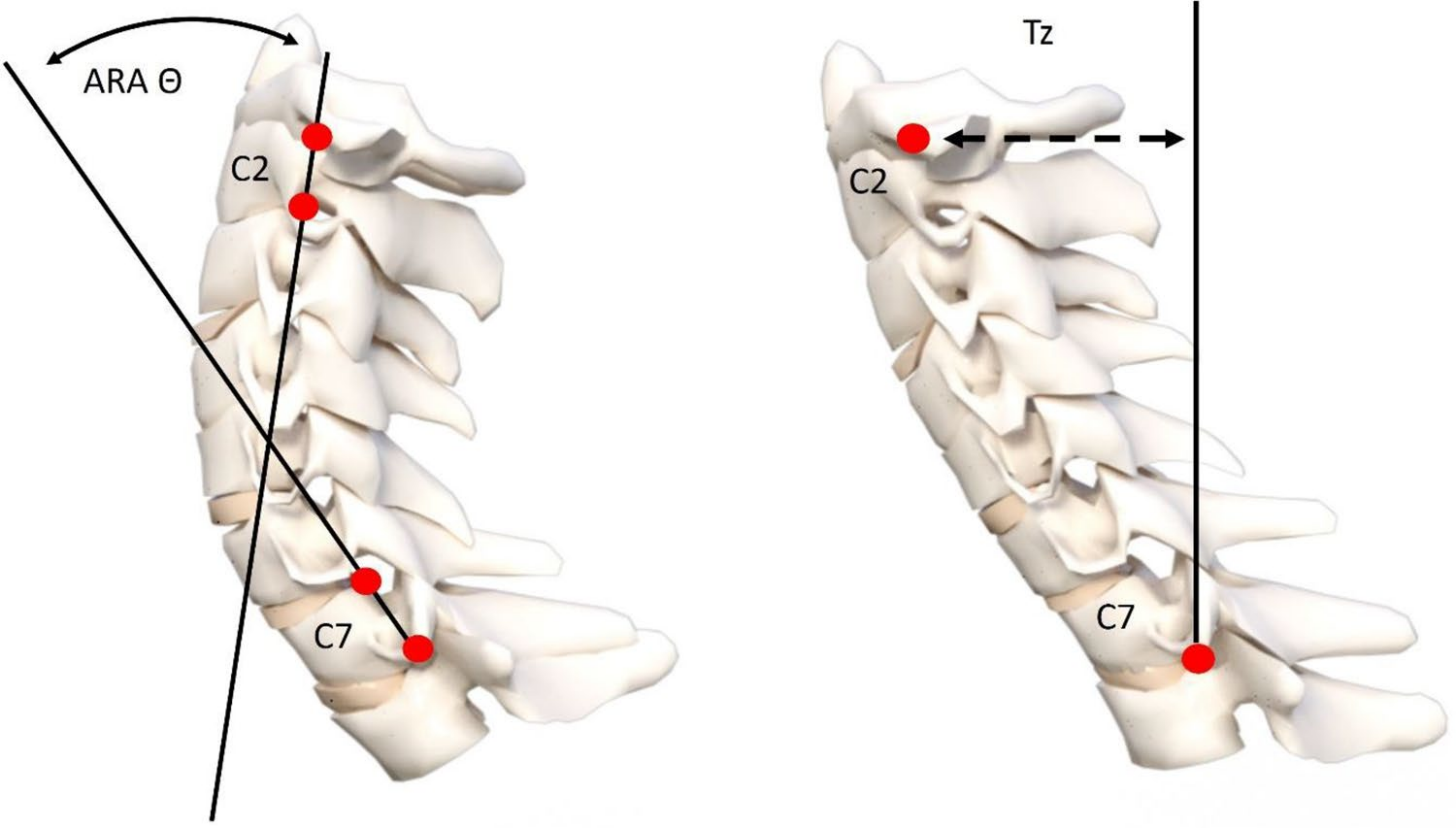
Radiographic measures: Absolute rotation angle (ARA) measurement of cervical lordosis from C2 to C7. Measurement of anterior head translation (AHT) using the horizontal offset of C2 relative to a vertical line originating at the posterior inferior body of C7^31,32^.

The outpatient databases of the two participating hospitals, Farouk hospital and Salam Hospital, were collected from September 2014 to April 2020, all eligible patients (N = 570) diagnosed with chronic nonspecific neck pain (CNSNP) were included in this investigation if they fulfilled the following criteria: CNSNP lasting for at least 3 months, aged 18–65 years, and able to read and speak English. Participants were screened prior to inclusion by measuring their lateral cervical spine radiographs for a magnitude of cervical lordosis using the ARA formed by two lines intersecting from the posterior body margins of C2–C7 and a forward head distance (AHT) measured as the horizontal displacement of the posterior superior body corner of C2 vertebra relative to a vertical line extending superiorly from the posterior inferior body corner of C7. Lateral cervical X-rays were obtained with the participant in an upright, neutral, standing posture with their right side against the x-ray cabinet. Participants with an ARA angle from 0° and less 20° and an AHT distance greater than 15 mm were considered eligible. The X-ray cut point for ARA was based on values previously reported to have good sensitivity and specificity^31^ and this measurement method has excellent examiner reliability^32^. Figure 6 demonstrates the radiographic alignment variables used herein.

Participant exclusion criteria were acute or subacute neck pain, a specific diagnosed cause of neck pain (e.g., systemic, rheumatic, neuromuscular diseases), central or peripheral neurological signs, cognitive impairment, spinal surgery, or physical therapy treatments in the 6 months prior to the baseline assessment, and participants without informed consent. Neither inclusion nor exclusion criteria were changed during the interventional timeframe. Data of all patients that fulfilled these inclusion criteria between September 2014 and April 2020 were extracted, and no patients were omitted (no selection bias). All eligible participants underwent a medical examination by an orthopedist, who excluded other specific causes of neck pain. All experimental protocols in this study were reviewed and approved by our Hospitals’ Ethics Committees (Far-REC-13-91 and REC-23 PT216). All experimental protocols were carried out following the guidelines of the World Medical Association Declaration of Helsinki. Informed consent was signed by each of the participants.

### Rehabilitation setting

All participants received a ‘Usual Care’ program in physical therapy, which is the current practice in physical therapy treatment at these two hospitals, without any influence or specific restriction from the researchers. The treatment was performed in the same two clinical settings.

### Usual care program

The Usual Care consisted of a multimodal approach with a combination of different techniques, such as trans-electrical nerve stimulation (TENS) and heat, soft tissue mobilization, functional exercises, and education. The treatment had a variation, based on specific patient needs and abilities, with a duration of 6 weeks, with 3 sessions a week, each with a duration of between 30 and 45 min.

### TENS and heat

The TENS and heat therapy were repeated with 3 sessions for 6 weeks. TENS was administered over the painful area with a frequency of 80 Hz, pulse width of 50 µs, intensity (mA) set at the individual's sensorial threshold, with a modulation up to 50% of variation frequency, symmetrical, and rectangular biphasic waveform. These parameters were configured for an optimal analgesic effect^43^. Moist hot packs (15 min) were applied prior to electrical stimulation. Moist hot packs were applied for 15 min before electrical stimulation.

### Soft tissue mobilization

Soft tissue mobilization targeted the upper quarter muscles, with the involved upper extremity positioned in abduction and external rotation to preload neural structures^44^. This soft tissue mobilization was repeated with 3 sessions for 6 weeks.

### Functional exercises

A functional and strengthening exercise program followed the protocol outlined in Harman et al.^45^. It included strengthening deep cervical flexors with chin tucks in supine lying, progressively lifting the head off the floor for varying durations. Shoulder retractors were strengthened initially while standing with a TheraBand, progressing to prone positions with weights. The standing position involved pinching the scapulae together without shoulder elevation or extension, held for at least six seconds before relaxing. Participants performed each progressive exercise for 2 weeks before advancing to the next difficulty level. Advancement to the next exercise occurred when participants could correctly complete 3 sets of 12 repetitions during consultation. The complete functional exercise program was to be repeated with 3 sessions for 6 weeks.

### Denneroll cervical traction orthotic (DCTO)

**Figure 7:**
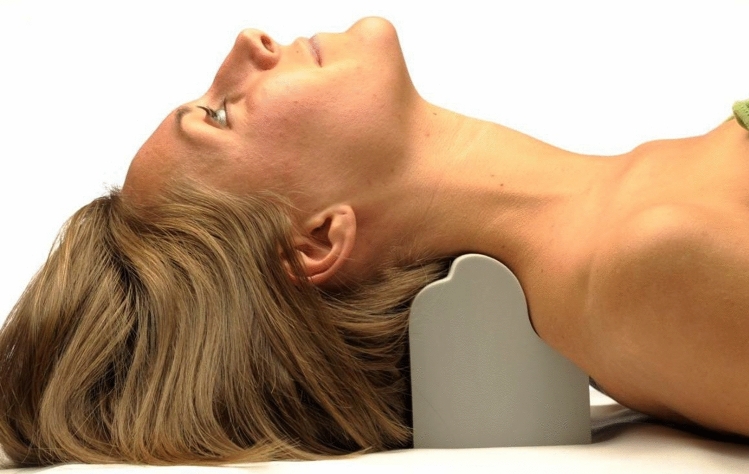
The Denneroll cervical traction orthotic (DCTO) for cervical extension traction (CET). Copyright CBP Seminars and reprinted with permission by author owner (DEH).

Using a protocol for CET^22^, participants were treated with a Denneroll cervical traction orthotic (DCTO) from Denneroll Industries (www.denneroll.com, accessed on Jan 1st, 2024) in Sydney, Australia. During the session, patients lie supine on the ground with extended legs and arms by their sides, and are encouraged to relax while lying on the Denneroll. The DCTO was placed on the ground and positioned in the posterior aspect of the neck, tailored to the specific area to be addressed based on radiographic identified curve apex and forward head posture amount, as illustrated in Fig. 7. Prior to use, participants were screened for tolerance to the neck extended and posterior head translation position on the device to ensure individual tolerance. The DCTO takes cervical spine segments near the apex of the curve to their end range of extension motion without causing hyper-extension of the skull relative to the torso.

Placement of the DCTO in the cervical spine is altered for each specific patient based on their radiographically determined malalignment and has 3 possible locations for device apex:

1. The upper cervical area (C2–C4). This position allows for upper cervical segment extension while providing minor anterior head translation (AHT).
2. The mid-cervical area (C4–C6). This position allows for mid-upper cervical extension while causing slight posterior head translation.
3. Lower cervical area (C6–T1). This position allows for lower to intermediate cervical segment extension while causing substantial posterior head translation.

All participants began with 3-min sessions on DCTO and were encouraged to increase the duration by 2–3 min each consecutive session until reaching the goal of 15–20 min each session. CET allows for viscoelastic plastic deformation of spinal ligaments as well as improving posture by initiating muscle and ligament creep. No standardized frequency and duration was forced; instead, we allowed variations based on participant tolerance. Treatment duration varied from 4 to 10 weeks, with frequencies varying from 3 to 6 times per week; some patients were allowed to use the DCTO at home pending needs and ability. Many external factors influenced these different protocols. The most significant challenge was the incorporation of this CET protocol along with an at home routine of exercise, making it challenging to precisely control these variables for all patients.

Safety and efficacy of CET is of paramount importance and these necessitate this protocol be performed under supervision. CET is a controlled, gradual process designed to enhance the cervical lordosis and alleviate symptoms associated with cervical spine disorders. The method involves careful positioning and monitoring to ensure that the traction is within therapeutic thresholds that do not overextend or strain the cervical spine. Monitoring for adverse effects was performed in our study, where participants were monitored for any signs of discomfort or exacerbation of symptoms throughout the duration of the treatment. This monitoring was conducted by trained physiotherapists who adjusted the traction force (size of the DCTO) and duration based on individual patient tolerance and response. Incidence of worsening symptoms was monitored throughout the study and any instances of increased pain or the development of new symptoms such as nerve root irritation was recorded. The data revealed that less than 2% of the participants experienced a transient increase in pain, only during the first two to three sessions which resolved without further intervention within 24 h. There were no reported cases of new or worsening nerve root symptoms directly attributable to the CET interventions. The safety protocols and guidelines for the CET protocol implemented in our study adhered to established clinical guidelines that specify that each patient begins with a low-intensity application, gradually increasing only as tolerated by the patient, ensuring a personalized approach to care^22,23,42^.

### Outcome measures

Functional, clinical, and demographic data of patients admitted to rehabilitation were obtained from their medical files. Demographic collected variables included age and BMI. While comprehensive clinical variables included cervical lordotic angle (ARA), AHT (mm), frequency of CET applications (number (No.) per week), duration of traction (No. of weeks), and compliance % (defined as the percentage of made vs. scheduled sessions). Functional variables included the neck disability index (NDI) which is a disability measure administered on study admission and at discharge.

#### Neck disability index (NDI)

The NDI is a widely used tool to assess the impact of neck pain on daily activities, providing a reliable and valid measure of disability associated with cervical spine conditions. It evaluates a patient's perceived limitations across various domains, aiding clinicians in understanding the functional impact of neck-related disorders^35^. The minimal detectable difference (MCD) and the minimal clinically important difference (MCID) for the NDI has been reported to be 6.9 and 5.5, respectively^35^. Accordingly, our data presents the percentage (%) of patients achieving a MCID using a threshold of 5.5 and 6 points on the NDI to be accurate in its findings.

#### Numerical pain rating score (NPRS)

The NPRS is a commonly employed reliable and valid pain assessment tool, allowing individuals to rate their pain intensity on an 11-point numerical scale. The scale ranges from 0 to 10, with 0 indicating no pain and 10 representing the highest level of pain^35^. The NPRS has an MDC of 2.6 and the MCID has been reported to be 1.5^35^. Therefore we chose the following more conservative threshold for the MCID of 2 points to indicate the percentage of patients who achieve the MCID at follow-up.

### Parameters used for the predictive model

The set of predicting variables included the following: Age, BMI, pretreatment cervical lordotic angle (ARA), AHT (mm), NDI, NPRS pain score, frequency of traction (No. per week), duration of traction (No. of weeks) and compliance percentage.

### Expected outcomes used for the predictive model

The post treatment ARA, post treatment NDI and post treatment NPRS pain score were used as output variables for the predictive model.

### Data analysis

The application of linear regression to forecast post-treatment outcomes represents a pivotal step in advancing our understanding of machine learning models' potential benefits and predictive capabilities in the context of healthcare interventions. This study employed linear regression to predict post-treatment outcomes among the 570 patients undergoing a multimodal program featuring the DCTO for application of CET. The primary objectives were to assess the feasibility of utilizing a machine learning model for predicting outcomes and to gain insights into the effectiveness of the DCTO CET procedure.

This study harnessed the power of computational tools and platforms to implement and analyze linear regression models. Python, a versatile programming language, served as the foundational platform for developing the models. The study extensively utilized sci-kit-learn, a robust machine learning library in Python, to implement linear regression algorithms. Furthermore, NumPy, a fundamental library for scientific computing in Python, played a crucial role in handling and processing the numerical data essential for training and evaluating the models.

## Data Availability

Data is available upon reasonable request from the corresponding author.
